# Methylation profiling of ductal carcinoma *in situ*and its relationship to histopathological features

**DOI:** 10.1186/s13058-014-0423-9

**Published:** 2014-10-21

**Authors:** Jia-Min B Pang, Siddhartha Deb, Elena A Takano, David J Byrne, Nicholas Jene, Alice Boulghourjian, Anne Holliday, Ewan Millar, C Soon Lee, Sandra A O’Toole, Alexander Dobrovic, Stephen B Fox

**Affiliations:** 1 Department of Pathology, Peter MacCallum Cancer Centre, St Andrews Place, Melbourne, 3002 VIC Australia; 2 Department of Pathology, University of Melbourne, Grattan Street, Parkville, Melbourne, 3010 VIC Australia; 3 Sir Peter MacCallum Department of Oncology, University of Melbourne, Grattan Street, Parkville, Melbourne, 3010 VIC Australia; 4 The Kinghorn Cancer Centre and Garvan Institute of Medical Research, Victoria Street, Darlinghurst, 2010 NSW Australia; 5 Department of Anatomical Pathology, South Eastern Area Pathology Service, St George Hospital, Gray Street, Kogarah, 2217 NSW Australia; 6 School of Medical Sciences, University of New South Wales, Kensington, 2052 NSW Australia; 7 Department of Tissue Pathology, Royal Prince Alfred Hospital, Missenden Road, Camperdown, 2050 NSW Australia; 8 Discipline of Pathology, School of Medicine, University of Western Sydney, Campbelltown, 2751 NSW Australia; 9 Cancer Pathology, Bosch Institute, University of Sydney, Sydney, 2006 NSW Australia; 10 Sydney Medical School, University of Sydney, Sydney, 2006 NSW Australia; 11 Translational Genomics & Epigenomics Laboratory, Ludwig Institute for Cancer Research, Olivia Newton-John Cancer & Wellness Centre, Heidelberg, 3084 VIC Australia

## Abstract

**Introduction:**

DNA methylation is a well-studied biomarker in invasive breast cancer, but its role in ductal carcinoma i*n situ* (DCIS) is less well characterized. The aims of this study are to assess the methylation profile in DCIS for a panel of well-characterized genes that are frequently methylated in breast cancer, to investigate the relationship of methylation with pathological features, and to perform a proof-of-principle study to evaluate the practicality of methylation as a biomarker in diagnostic DCIS material.

**Methods:**

Promoter CpG island methylation for a panel of 11 breast cancer-related genes was performed by methylation-sensitive high resolution melting (MS-HRM). Formalin-fixed, paraffin-embedded (FFPE) biopsies from 72 samples of pure DCIS (DCIS occurring in the absence of synchronous invasive carcinoma), 10 samples of mixed DCIS (DCIS adjacent to invasive carcinoma), and 18 samples of normal breast epithelium adjacent to a DCIS lesion were micro-dissected prior to DNA extraction.

**Results:**

Methylation was seen for all the tested genes except *BRCA1. RASSF1A* was the most frequently methylated gene (90% of DCIS samples) and its methylation was associated with comedo necrosis (p = 0.018). Cluster analysis based on the methylation profile revealed four groups, the highly methylated cluster being significantly associated with high nuclear grade, *HER2* amplification, negative estrogen receptor (ER) α status, and negative progesterone receptor (PgR) status, (p = 0.038, p = 0.018, p <0.001, p = 0.001, respectively). Methylation of *APC* (p = 0.017), *CDH13* (p = 0.017), and *RARβ* (p <0.001) was associated with negative ERα status. Methylation of *CDH13* (p <0.001), and *RARβ* (p = 0.001) was associated with negative PgR status. Methylation of *APC* (p = 0.013) and *CDH13* (p = 0.026) was associated with high nuclear grade. Methylation of *CDH13* (p = 0.009), and *RARβ* (p = 0.042) was associated with *HER2*-amplification.

**Conclusions:**

DNA methylation can be assessed in FFPE-derived samples using suitable methodologies. Methylation of a panel of genes that are known to be methylated in invasive breast cancer was able to classify DCIS into distinct groups and was differentially associated with phenotypic features in DCIS.

**Electronic supplementary material:**

The online version of this article (doi:10.1186/s13058-014-0423-9) contains supplementary material, which is available to authorized users.

## Introduction

Ductal carcinoma *in situ* (DCIS), a non-invasive form of breast cancer and a non-obligate precursor of invasive carcinoma of the breast, has both morphological and biological heterogeneity. Current markers of poor prognosis to help select the use of adjuvant therapies are largely based on clinical and histopathological parameters, and include young age, large tumour size, high nuclear grade, presence of comedo necrosis, negative hormone receptor status, and *HER2* amplification [1],[2]. However, these clinicopathological features are insufficient in predicting which patients will experience recurrence of DCIS or progress to invasive carcinoma [1]-[3]. Therefore, more informative and robust prognostic markers are required, which also need to be compatible with small amounts of often degraded, formalin-fixed, paraffin-embedded (FFPE)-derived DNA, as typically only a sparse amount of material is available for analysis from DCIS lesions.

DNA methylation is an epigenetic modification where a methyl group is added to the 5-carbon position of cytosine and is a mechanism of modulating gene expression. Alterations in methylation patterns in cancer are characterized by global hypomethylation and gene-specific promoter hypermethylation. Promoter hypermethylation may result in gene silencing, and in cancer this can be a mechanism of tumour suppressor gene inactivation. Promoter methylation frequently follows a tumour-specific pattern and has been reported to be a useful biomarker in several types of cancer, including invasive breast cancer [4]. Several studies have linked methylation of specific genes to DCIS phenotypes, including *APC*[5], *CDH1*[6], *FOXC1*[7],[8], *GSTP1*[7]-[9], *RARβ*[5], and *RASSF1A*[7],[8]. However, most of these studies have examined methylation in a small number of pure DCIS cases [5],[7]-[10], or combined invasive breast cancer cases together with DCIS cases to establish the relationship between methylation and phenotype [7],[8]. Therefore, the true frequency and utility of DNA methylation biomarkers in DCIS has yet to be established [11].

The aims of this study were to document the frequency and level of methylation of a panel of eleven breast cancer-related genes to determine whether these methylated genes are associated with histopathological parameters. These genes were chosen because methylation of the genes have previously been identified as important in invasive breast cancer by The Cancer Genome Atlas Network (TCGA) [12] and/or associated with prognosis in DCIS (*APC*[5]*, CDH1*[6]*, FOXC1*[7]*, GSTP1*[9]*, RARβ*[5]*, RASSF1A*[7]) or invasive carcinoma (*BRCA1*[13],[14]*, CDH13*[15]*, MAL*[16]*, TWIST1*[17]*, WIF1*[18]). The goal of the study was to improve our understanding of methylation in *in situ* breast cancer, to understand its relation to important histopathological variables and conduct a proof-of-principle study to assess the potential of methylation status as a biomarker in patients with DCIS.

## Materials and methods

### Patients and samples

FFPE blocks were obtained from primary DCIS cases from Peter MacCallum Cancer Centre and Royal Prince Alfred Hospital. Approval for the project was obtained from the ethics committees of Peter MacCallum Cancer Centre (project number 02/26 and 10/16) and Royal Prince Alfred Hospital (project HREC/11/RPAH/126), including a waiver of consent for the use of archival material for research. A total of 72 pure DCIS samples (DCIS occurring in the absence of synchronous invasive carcinoma), 10 mixed DCIS samples (DCIS adjacent to invasive carcinoma) and 18 samples of normal breast epithelium (including 16 normal samples matched to DCIS from the same paraffin block) were obtained from 79 patients (69 patients with pure DCIS and 10 patients with mixed DCIS). Patient flow in the study is shown in Additional file 1.

Patient and sample characteristics are summarized in Table 1. All patients were female. The median age of the cohort was 54 years (range 29 to 82 years), and median tumour size was 32.8 mm (range 5.0 to 145.0 mm).

**Table 1:** **Characteristics of the cohort**

Feature		Pure ductal carcinoma***in situ***(n = 72)	Mixed ductal carcinoma***in situ***(n = 10)	All ductal carcinoma***in situ***(n = 82)
Age	Median, years	54.0	53.0	54.0
	Range, years	29 to 82	42 to 67	29 to 82
	No data	14/69	1/10	15/79
Lesion size	Median, mm	32.0	40.0	32.8
	Range, mm	5 to 145	11 to 103	5 to 145
Nuclear grade	High	35/72 (48.6%)	4/10 (40%)	39/82 (47.6%)
	Intermediate	31/72 (43.1%)	6/10 (60%)	37/82 (45.1%)
	Low	6/72 (8.3%)	0/10 (0%)	6/82 (7.3%)
Predominant architectural pattern	Solid	37/72 (51.4%)	10/10 (100%)	47/82 (57.3%)
	Cribriform	17/72 (23.6%)	0/10 (0%)	17/82 (20.7%)
	Micropapillary	11/72 (15.3%)	0/10 (0%)	11/82 (13.4%)
	Clinging	4/72 (5.6%)	0/10 (0%)	4/82 (4.9%)
	Cancerisation of lobules	2/72 (2.8%)	0/10 (0%)	2/82 (2.4%)
	Papillary	1/72 (1.4%)	0/10 (0%)	1/82 (1.2%)
Comedo necrosis	Present	29/72 (40.3%)	6/10 (60%)	35/82 (42.7%)
	Absent	43/72 (59.7%)	4/10 (40%)	47/82 (57.3%)
Estrogen receptor status	Positive	48/67 (71.6%)	7/8 (87.5%)	55/75 (73.3%)
	Negative	19/67 (28.4%)	1/8 (12.5%)	20/75 (26.7%)
	No data	5/72	2/10	7/82
Progesterone receptor status	Positive	40/67 (59.7%)	3/8 (37.5%)	43/75 (57.3%)
	Negative	27/67 (40.3%)	5/8 (62.5%)	32/75 (42.7%)
	No data	5/72	2/10	7/82
*HER2* amplification	Amplified	20/67 (29.9%)	2/8 (25%)	22/75 (29.3%)
	Non-amplified	47/67 (70.1%)	6/8 (75%)	53/75 (70.7%)
	No data	5/72	2/10	7/82
Intrinsic subtype	Luminal	38/67 (56.7%)	6/8 (75%)	44/75 (58.7%)
	HER2	20/67 (29.9%)	2/8 (25%)	22/75 (29.3%)
	Basal	2/67 (3.0%)	0/8 (0%)	2/75 (2.6%)
	Negative	7/67 (10.4%)	0/8 (0%)	7/75 (9.3%)
	No data	5/72	2/10	7/82

H&E-stained sections of the FFPE blocks used for DNA extraction were reviewed by a pathologist. The nuclear grade of DCIS was determined according to the guidelines described in the *WHO Classification of Tumours of the Breast, 4*^*th*^*edition*[19]. Immunohistochemical (IHC) staining for estrogen receptor (ER)α, progesterone receptor (PgR), and cytokeratin 5 (CK5) and *HER2* silver *in situ* hybridization (SISH) were performed as previously described [20],[21]. Tumours were considered to be ERα-positive and PgR-positive if at least 10% of tumour cells showed nuclear staining, and were considered *HER-2* amplified if there were at least six dots or large clusters of dots in the tumour nuclei [22].

Tumours were classified into intrinsic subtypes by IHC staining of tissue microarrays (TMA), based on criteria for invasive carcinomas of Nielsen *et al*. [23] and Cheang *et al*. [24], and similar to those previously used in DCIS [25]-[27]. Tumours exhibiting ERα or PgR positivity in the absence of *HER2* amplification were considered of luminal subtype, human epidermal growth factor receptor-2 (HER2) subtype consisted of tumours with *HER2* amplification, regardless of ERα and PgR status, basal-like subtype consisted of triple negative (ERα negative, PgR negative, *HER2* non-amplified) tumours with any degree of CK5 membranous staining, and negative subtype tumours consisted of triple-negative tumours without CK5 staining.

### DNA preparation and bisulfite modification

Areas of DCIS and adjacent normal breast epithelium were needle micro-dissected with the aid of a dissecting microscope from up to 72 methyl green-stained 7 μm-thick parallel sections. No invasive carcinoma was present in the available paraffin block of the ten cases of mixed DCIS. Genomic DNA was extracted from the micro-dissected tissues using the QIAamp DNA Blood Mini Kit (Qiagen, Hilden, Germany) as previously described [28]. Five hundred nanograms of genomic DNA were bisulfite-modified using the MethylEasy Xceed Rapid DNA Bisulphite Modification Kit (Human Genetic Signatures, Sydney, Australia) according to the manufacturer’s instructions. The bisulfite-modified DNA was eluted to achieve a final concentration of 10 ng/μL. Universal Methylated DNA (CpGenome Universal Methylated DNA, Millipore, Billerica, MA, USA) and whole genome amplified (WGA) peripheral blood mononuclear DNA (Ready-To-Go GenomiPhi V3 DNA Amplification Kit, GE Healthcare, Buckinghamshire, UK) were bisulfite-modified as above and used as fully methylated (100%) and unmethylated (0%) controls, respectively. Methylation standards (50%, 25%, and 10% methylated) were prepared by diluting fully methylated DNA into unmethylated DNA.

### Methylation-sensitive high resolution melting (MS-HRM)

Methylation analysis was performed using MS-HRM, a robust, real-time PCR-based methodology which allows semiquantitative assessment of homogeneous methylation and identification of heterogeneous methylation [29],[30]. This method distinguishes between methylated and unmethylated templates based on melting profiles conferred by sequence alterations as a result of bisulfite modification. Methylated templates contain more cytosines compared with unmethylated templates after bisulfite conversion and therefore melt at a higher temperature. Heterogenous methylated templates are identified from complex melting patterns that arise as a consequence of heteroduplex formation [31]. Examples are shown in Additional file 2.

MS-HRM primers were designed according to guidelines previously described [32]. Primer sequences are listed in Additional file 3. The PCR reaction mixture consisted of 1 × PCR buffer (Qiagen, Hilden, Germany), 1.5 to 3.0 mm MgCl_2_ (Qiagen), 200 μM dNTP mix (Fisher Biotech, Perth, Australia), 200 to 400 nmol/L forward and reverse primers, 1 × SYTO9 intercalating dye (Life Technologies, Carlsbad, CA, USA), 0.5U HotstarTaq polymerase (Qiagen), and 10 to 20 ng of bisulfite modified DNA, in a total reaction volume of 20 μL. PCR amplification and high-resolution melting were performed using the Rotor-Gene Q (Qiagen). PCR and high resolution melting conditions are listed in Additional file 4. All assays were performed in duplicate with fully methylated, 50%, 25%, 10%, and fully unmethylated DNA standards and non-template and non-bisulfite-modified genomic DNA controls.

Homogeneous methylation was scored as low (<10%), moderate (10% to <50%), and high (≥50%) level methylation. Heterogeneous methylation was scored as low-level or high-level heterogeneous methylation depending on the sample profile extension into the fully methylated profile (examples in Additional file 2). To compensate for non-specific background methylation, only samples with moderate- and high-level homogeneous methylation or high-level heterogeneous methylation were considered methylated. The average methylation index (AMI) for each sample was also calculated, which is similar to the cumulative methylation index described by Fackler *et al*. [33], but normalized for the number of genes assessed. Methylation levels at or close to 0%, 10%, 25%, 50%, and 100% were scored as such. Low heterogeneous methylation and <10% homogeneous methylation were scored as 0%, and 10% to 25%, 25% to 50%, and 50% to 100% methylation were scored as 18%, 38%, and 75%, methylation respectively. High-level heterogeneous methylation, which is not possible to quantify, was assigned an arbitrary score of 25% methylation.

### Statistical analysis

Comparisons of continuous data between two groups, and more than two groups were evaluated by the Mann-Whitney *U*-test and the Kruskal-Wallis test, respectively. Fisher’s exact probability test was used to assess 2 × 2 contingency tables and the χ^2^ test for independence was used for variables with three or more categories. For each comparison, a two-tailed *P*-value of 0.05 or less was considered to be statistically significant. All statistical analyses were performed using IBM SPSS version 22.0 (IBM Corporation, Armonk, NY, USA). Unsupervised hierarchical cluster analysis with average linkage was performed giving equal weighting to all genes and samples, with the exception of *BRCA1* which was universally unmethylated and therefore removed from the analysis. The samples were filtered to include only those with methylation data for at least nine of the eleven genes. The cluster analysis was performed and heat map generated using Gene Cluster 3.0 and TreeView 1.60, respectively (Michael Eisen, University of California, USA). Histograms were generated using GraphPad Prism 6 (La Jolla, CA, USA).

## Results

### Histopathological features of DCIS

There were 47.6%, 45.1%, and 7.3% of DCIS samples of high, intermediate and low nuclear grade, respectively. The most frequent architectural pattern was solid type (47/82, 57.3%), followed by cribriform (17/82, 20.7%), and micropapillary (11/82, 13.4%) patterns. The remainder of the samples showed clinging DCIS (4/82, 4.9%), cancerisation of lobules (2/82, 2.4%) and papillary DCIS (1/82, 1.2%). Comedo-type necrosis was present in 42.7% (35/82) of DCIS samples. Fifty-five DCIS samples (55/75, 73.3%) were ERα-positive, and 43 samples (43/55, 57.3%) were PgR-positive. *HER2* amplification was present in 22 samples (22/75, 29.3%). Luminal subtype accounted for 58.7% of samples (44/75), HER2 subtype 29.3% (22/75), negative subtype 9.3% (7/75), and basal-like subtype 2.7% (2/75).

### Methylation status in normal, pure DCIS, and mixed DCIS samples

**Figure 1:**
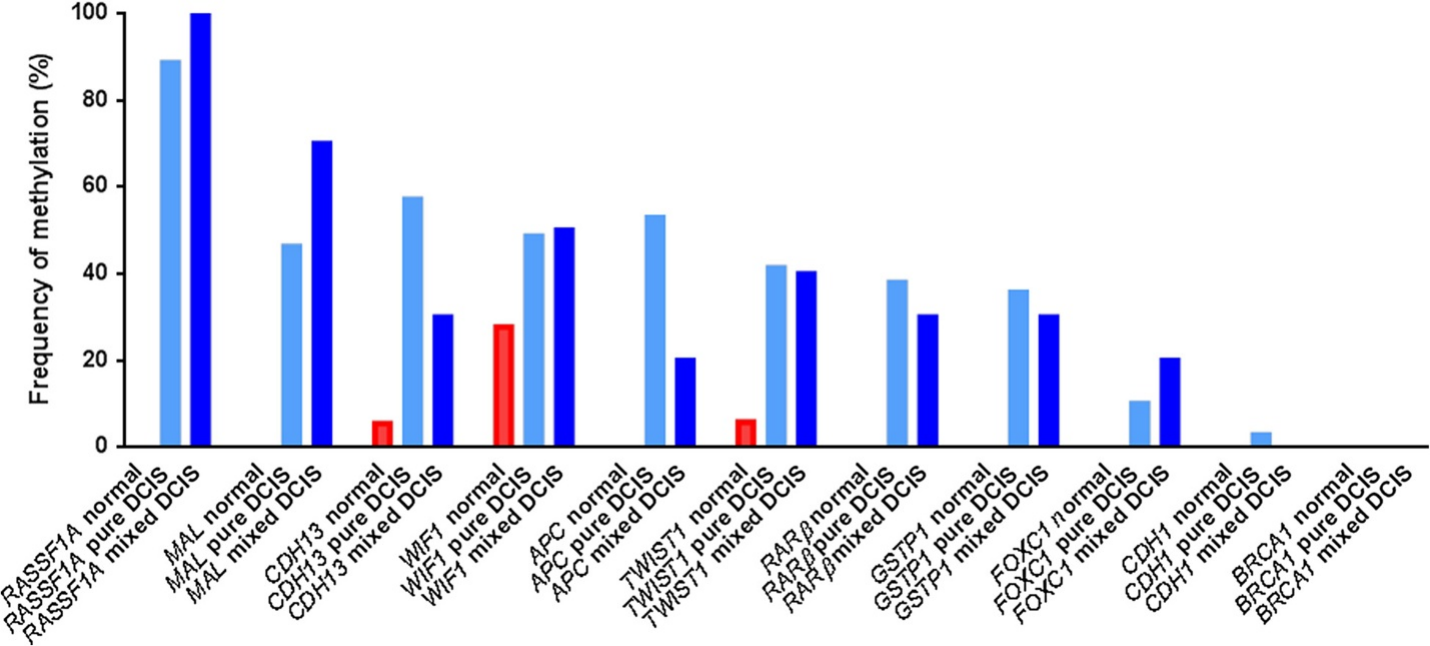
**Frequency of methylation of each gene by sample type.** DCIS, ductal carcinoma *in situ*.

DCIS samples had a significantly greater number of genes methylated (a median of 4 genes methylated, range 0 to 8 genes) compared with adjacent normal breast epithelium samples (median 0 genes methylated, range 0 to 2 genes) (*P* <0.001). There was no significant difference in the number of genes methylated per sample between pure DCIS (median 4.5 genes, range 0 to 8 genes) and mixed DCIS samples (median 3 genes, range 2 to 8 genes) (*P* = 0.87). Among all DCIS, *RASSF1A* methylation was present in 90% (72/80) of samples, *CDH13* in 53.8% (43/80), *MAL* in 49.4% (39/79), *APC* in 48.8% (39/80), *WIF1* in 48.8% (39/80), *GSTP1* in 47.5% (38/80), *TWIST1* in 40.7% (33/81), *RARβ* in 37% (30/81), and *FOXC1* methylation in 11.3% (9/80) of samples. *CDH1* methylation was rare (2.5%, 2/79) (Table 2, Figure 1).

**Table 2:** **Frequency and level of methylation of genes by sample type**

		Negative for methylation			Positive for methylation				
		Frequencies (%)							
Gene	Sample type	No methylation	Low heterogenous methylation	Low homogenous methylation	High heterogenous methylation	Moderate homogenous methylation	High homogenous methylation	Total positive for methylation	No data
***APC***	Normal	15/15 (100)	0/15 (0)	0/15 (0)	0/15 (0)	0/15 (0)	0/15 (0)	0/15 (0)	3
	Pure ductal carcinoma *in situ* (DCIS)	29/70 (41.4)	0/70 (0)	4/70 (5.7)	4/70 (5.7)	27/70 (38.6)	6/70 (8.6)	37/70 (52.9)	2
	Mixed DCIS	7/10 (70)	1/10 (10)	0/10 (0)	0/10 (0)	1/10 (10)	1/10 (10)	2/10 (20)	0
***BRCA1***	Normal	17/17 (100)	0/17 (0)	0/17 (0)	0/17 (0)	0/17 (0)	0/17 (0)	0/17 (0)	1
	Pure DCIS	70/70 (100)	0/70 (0)	0/70 (0)	0/70 (0)	0/70 (0)	0/70 (0)	0/70 (0)	2
	Mixed DCIS	10/10 (100)	0/10 (0)	0/10 (0)	0/10 (0)	0/10 (0)	0/10 (0)	0/10 (0)	0
***CDH1***	Normal	16/17 (94.1)	0/17 (0)	1/17 (5.9)	0/17 (0)	0/17 (0)	0/17 (0)	0/17 (0)	1
	Pure DCIS	62/69 (89.9)	0/69 (0)	5/69 (7.2)	2/69 (2.9)	0/69 (0)	0/69 (0)	2/69 (2.9)	3
	Mixed DCIS	9/10 (90)	0/10 (0)	1/10 (10)	0/10 (0)	0/10 (0)	0/10 (0)	0/10 (0)	0
***CDH13***	Normal	16/18 (88.9)	0/18 (0)	1/18 (5.6)	0/18 (0)	1/18 (5.6)	0/18 (0)	1/18 (5.6)	0
	Pure DCIS	30/70 (42.9)	0/70 (0)	0/70 (0)	32/70 (45.7)	6/70 (8.6)	2/70 (2.9)	40/70 (57.1)	2
	Mixed DCIS	6/10 (60)	1/10 (10)	0/10 (0)	2/10 (20)	0/10 (0)	1/10 (10)	3/10 (30)	0
***FOXC1***	Normal	16/17 (94.1)	0/17 (0)	1/17 (5.9)	0/17 (0)	0/17 (0)	0/17 (0)	0/17 (0)	1
	Pure DCIS	59/70 (84.3)	1/70 (1.4)	3/70 (4.3)	6/70 (8.6)	1/70 (1.4)	0/70 (0)	7/70 (10)	2
	Mixed DCIS	7/10 (70)	1/10 (10)	0/10 (0)	1/10 (10)	1/10 (10)	0/10 (0)	2/10 (20)	0
***GSTP1***	Normal	15/16 (93.8)	0/16 (0)	1/16 (6.3)	0/16 (0)	0/16 (0)	0/16 (0)	0/16 (0)	2
	Pure DCIS	30/70 (42.9)	4/70 (5.7)	1/70 (1.4)	10/70 (14.3)	7/70 (10)	18/70 (25.7)	35/70 (50)	2
	Mixed DCIS	7/10 (70)	0/10 (0)	0/10 (0)	1/10 (10)	1/10 (10)	1/10 (10)	3/10 (30)	0
***MAL***	Normal	14/15 (93.3)	0/15 (0)	1/15 (6.7)	0/15 (0)	0/15 (0)	0/15 (0)	0/15 (0)	3
	Pure DCIS	31/69 (44.9)	5/69 (7.2)	1/69 (1.4)	32/69 (46.3)	0/69 (0)	0/69 (0)	32/69 (46.3)	3
	Mixed DCIS	3/10 (30)	0/10 (0)	0/10 (0)	7/10 (70)	0/10 (0)	0/10 (0)	7/10 (70)	0
***RARβ***	Normal	17/17 (100)	0/17 (0)	0/17 (0)	0/17 (0)	0/17 (0)	0/17 (0)	0/17 (0)	1
	Pure DCIS	43/71 (60.6)	0/71 (0)	1/71 (1.4)	1/71 (1.4)	16/71 (22.5)	10/71 (14.1)	27/71 (38.0)	1
	Mixed DCIS	7/10 (70)	0/10 (0)	0/10 (0)	0/10 (0)	0/10 (0)	3/10 (30)	3/10 (30)	0
***RASSF1A***	Normal	15/18 (83.3)	1/18 (5.6)	2/18 (11.1)	0/18 (0)	0/18 (0)	0/18 (0)	0/18 (0)	0
	Pure DCIS	6/70 (8.6)	0/70 (0)	2/70 (2.9)	0/70 (0)	32/70 (45.7)	30/70 (42.9)	62/70 (88.6)	2
	Mixed DCIS	0/10 (0)	0/10 (0)	0/10 (0)	0/10 (0)	4/10 (40)	6/10 (60)	10/10 (100)	0
***TWIST1***	Normal	16/17 (94.1)	0/17 (0)	0/17 (0)	1/17 (5.9)	0/17 (0)	0/17 (0)	1/17 (5.9)	1
	Pure DCIS	34/71 (47.9)	4/71 (5.6)	4/71 (5.6)	26/71 (36.6)	3/71 (4.2)	0/71 (0)	29/71 (41.4)	1
	Mixed DCIS	6/10 (60)	0/10 (0)	0/10 (0)	3/10 (30)	0/10 (0)	1/10 (10)	4/10 (40)	0
***WIF1***	Normal	13/18 (72.2)	0/18 (0)	0/18 (0)	5/18 (27.8)	0/18 (0)	0/18 (0)	5/18 (27.8)	0
	Pure DCIS	31/70 (44.3)	0/70 (0)	5/70 (7.1)	31/70 (44.3)	3/70 (4.3)	0/70 (0)	34/70 (48.6)	2
	Mixed DCIS	4/10 (40)	0/10 (0)	1/10 (10)	3/10 (30)	2/10 (20)	0/10 (0)	5/10 (50)	0

There was no difference in methylation in samples from patients younger than 50 years compared with older patients (*P* >0.05, data not shown). In addition, there was no difference in methylation between small DCIS tumours (<20 mm [1]) and larger tumours (*P* >0.05, data not shown). No *BRCA1* methylation was present in any of the DCIS samples (Table 2).

In the normal breast epithelium samples, only *WIF1* methylation (27.8%, 5/18), *CDH13* (5.6%, 1/18), and *TWIST1* (5.9%, 1/17) methylation were identified (Table 2, Figure 1). For all methylated normal samples, the corresponding DCIS tumour also was methylated for the same gene, apart from one case (sample S25) where the normal tissue showed heterogeneous methylation for *WIF1* while the DCIS was unmethylated (Additional file 5).

### Association of methylation with DCIS phenotype

Methylation of *APC* and *CDH13* was significantly associated with high nuclear grade (*P* = 0.013 and *P* = 0.026 respectively). *RASSF1A* methylation was significantly correlated with comedo-type necrosis (*P* = 0.018). None of the other genes assessed showed an association with nuclear grade or presence of comedo-type necrosis (*P* >0.05). No association was found between methylation of the genes in the panel and DCIS architectural pattern (*P* >0.05) (Table 3).

**Table 3:** **Relationship between methylation and phenotypic features of digital carcinoma** ***in situ***

	Methylated gene										
	***APC***	***BRCA1***	***CDH1***	***CDH13***	***FOXC1***	***GSTP1***	***MAL***	***RARB***	***RASSF1A***	***TWIST1***	***WIF1***
High nuclear grade	64.9% HG, 34.9% non-HG ***P*** **= 0.013**	*P* = 1.000	*P* = 1.000	67.6% HG, 41.9% non-HG ***P*** **= 0.026**	*P* = 0.073	*P* = 0.178	*P* = 1.000	*P* = 0.106	*P* = 0.275	*P* = 1.000	*P* = 0.116
Architectural pattern	*P* = 0.620	*P* = 1.000	*P* = 0.907	*P* = 0.256	*P* = 0.596	*P* = 0.519	*P* = 0.399	*P* = 0.365	*P* = 0.254	*P* = 0.311	*P* = 0.402
Comedo necrosis	*P* = 0.175	*P* = 1.000	*P* = 0.503	*P* = 0.500	*P* = 0.159	*P* = 0.498	*P* = 0.652	*P* = 1.000	100% CN, 82.6% non-CN ***P*** **= 0.018**	*P* = 0.253	*P* = 0.824
ERα	41.5% ERα+, 75% ERα- ***P*** = **0.017**	*P* = 1.000	*P* = 0.481	47.2% ERα+, 80% ERα- ***P*** **= 0.017**	*P* = 0.246	*P* = 0.794	*P* = 0.111	27.8% ERα+, 75% ERα-***P*** **<0.001**	*P* = 0.336	*P* = 0.604	*P* = 0.794
PgR	*P* = 0.236	*P* = 1.000	*P* = 1.000	37.2% PgR+, 83.3% PgR- ***P*** **<0.001**	*P* = 0.473	*P* = 1.000	*P* = 0.230	23.3% PgR+, 64.5% PgR- ***P*** **= 0.001**	*P* = 1.000	*P* = 1.000	*P* = 0.346
*HER2*	*P* = 0.607	*P* = 1.000	*P* = 1.000	81.0% *HER2,* 46.2% non-HER2 ***P*** **= 0.009**	*P* = 0.711	*P* = 0.302	*P* = 0.305	59.1% *HER2,* 32.7% non-HER2 ***P*** **= 0.042**	*P* = 0.173	*P* = 0.446	*P* = 0.607
Intrinsic subtype	*P* = 0.184	*P* = 1.000	*P* = 0.252	Basal 50%, HER2 81.0%, Luminal 39.5%, Negative 85.7% ***P*** **= 0.006**	*P* = 0.136	*P* = 0.08	*P* = 0.368	Basal 100%, HER2 59.1%, Luminal 23.3%, Negative 71.4% ***P*** **= 0.003**	*P* = 0.113	*P* = 0.597	*P* = 0.111

HG, high nuclear grade, CN, comedo necrosis. Detailed results of all analyses are tabulated in Additional file 6.

*APC, CDH13* and *RARβ* methylation was significantly associated with ERα-negative DCIS (*P* = 0.017, *P* = 0.017 and *P* <0.001 respectively) and *CDH13* and *RARβ* methylation were also significantly associated with PgR-negative DCIS (*P* <0.001 and *P* = 0.001). *HER2* amplification in DCIS tumours was associated with methylation of *CDH13* (*P* = 0.009), and *RARβ* (*P* = 0.042). *CDH13* and *RARβ* methylation were also significantly associated with intrinsic subtype of DCIS (*P* = 0.006 and *P* = 0.003 respectively). None of the other genes assessed showed a significant relationship with hormone receptor status, *HER2* amplification, or intrinsic subtype (*P* >0.05) (Table 3).

**Figure 2:**
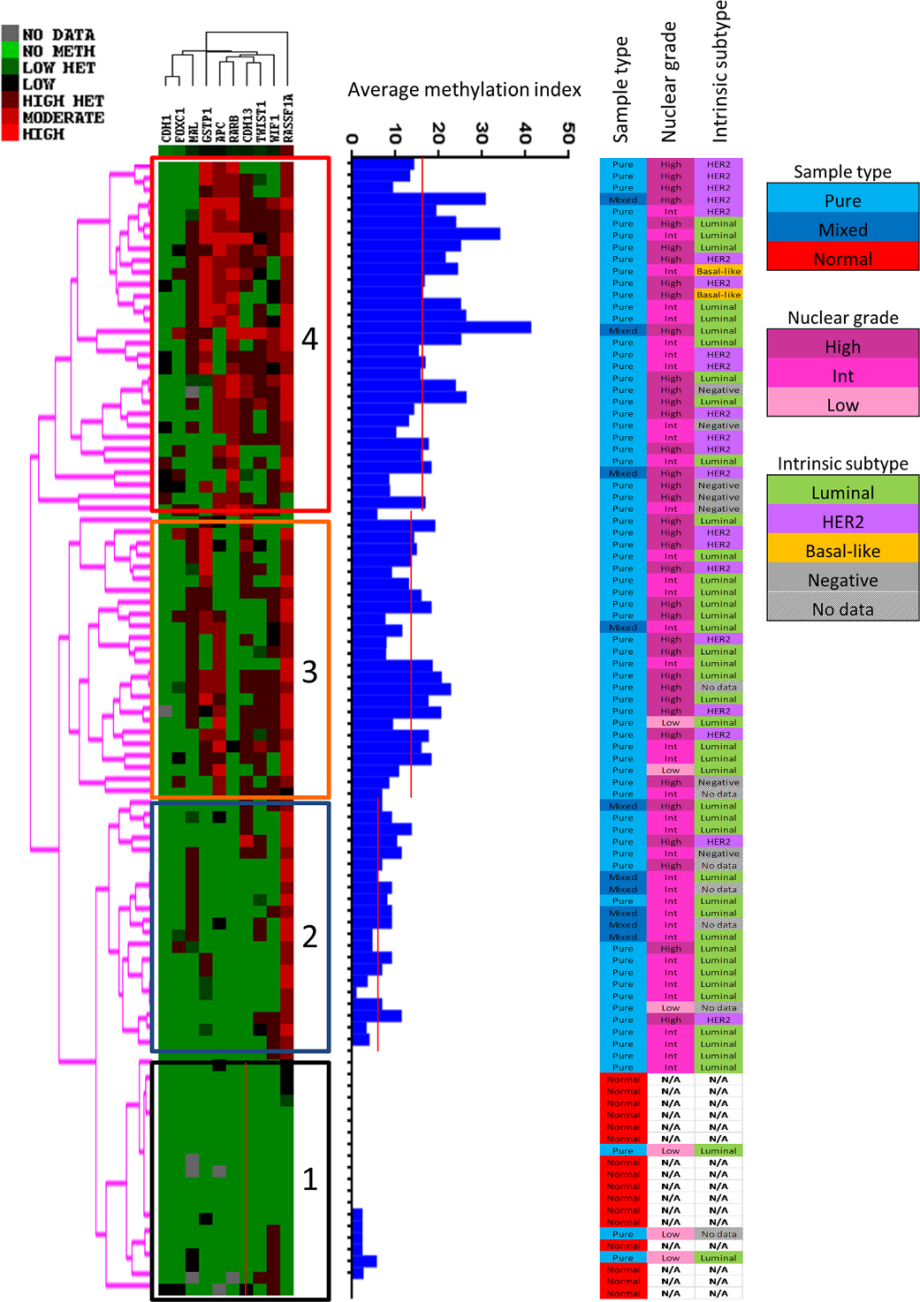
**Heat map generated from unsupervised hierarchical cluster analysis.** The ductal carcinoma *in situ* samples can be separated into a minimal methylation group (group 1) which also contains normal samples, a low methylation group (group 2), an intermediate group (group 3) and a high methylation group (group 4). The average methylation (AMI) index of each sample is indicated in the histogram, the red line shows the median AMI for each cluster.

Unsupervised hierarchical cluster analysis based on methylation profile of this panel of genes demonstrated four main groups (Figure 2). Cluster 1 samples had minimal methylation, cluster 2 samples were characterized by *RASSF1A* methylation, cluster 3 showed *APC, CDH13,* and *GSTP1* methylation in addition to *RASSF1A,* and cluster 4 samples were extensively methylated with the addition of *RARβ* and *WIF1* methylation. As expected, the increasing methylation is reflected in the median AMI of the clusters, the median AMI of clusters 1, 2, 3, and 4, being 0, 7.4, 14.5, and 17.2 respectively (*P* <0.001). Cluster-4 samples were significantly associated with high nuclear grade, *HER2* amplification, negative ERα status, negative PR status, and non-luminal intrinsic subtype (*P* = 0.038, *P* = 0.018, *P* <0.001, *P* = 0.001, and *P* <0.001, respectively) compared with DCIS samples in the other clusters (Additional file 6). Nuclear grade remained a distinguishing feature of the other clusters, with cluster 3 containing significantly more high nuclear grade samples compared with clusters 1 and 2 (*P* = 0.004), and cluster 1 containing significantly more low-grade DCIS samples compared with cluster 2 (*P* = 0.013) (Additional file 6).

## Discussion

In this study, we assessed methylation of a panel of breast cancer-associated genes in a large cohort of DCIS cases, and assessed the relationship of methylation with clinicopathological features. As expected, methylation was rarely present in adjacent morphologically normal breast epithelium. Interestingly, *CDH13*, *TWIST1*, and *WIF1* were methylated in a small number of normal epithelium samples. These were also methylated in the adjacent DCIS in all but one case (*WIF1* in sample S25). Several of the normal samples had more than one methylated gene. This is consistent with the possibility that methylation of these genes is an early change, and/or possibly reflects a methylation field effect in DCIS, which has been previously reported [34]. It would therefore be of interest to further compare methylation of *CDH13*, *TWIST1*, and *WIF1* in DCIS, normal epithelium adjacent to DCIS, and normal epithelium from healthy breast (such as the contralateral breast) in an independent cohort to determine whether methylation of these genes observed in normal samples is a disease-related event.

No significant difference was present between pure DCIS and mixed DCIS samples. Although the absence of a difference in methylation between pure and mixed DCIS may reflect the small number of mixed DCIS samples in this cohort, it is likely that aberrant DNA methylation is an early event in breast cancer progression, with gradual accumulation of methylation changes from epithelium of normal appearance to non-malignant epithelial lesions to DCIS, while the transition from DCIS to invasive carcinoma is less likely to rely on methylation, at least not for the genes studied here [5],[7],[9],[35],[36].

Of particular interest was the total absence of *BRCA1* methylation in this cohort. Given a frequency of approximately 20% *BRCA1* methylation in invasive carcinoma [37], this suggests that tumours driven by *BRCA1* methylation either rarely pass through a DCIS phase or have an exceedingly rapid transit through one, a notion supported by observation that DCIS is rare in carriers with *BRCA1* germline mutations [38],[39].

Cluster analysis based on methylation profile divided our DCIS cohort into four groups, which were phenotypically distinguished by nuclear grade, and in particular, the high-methylation cluster (cluster 4), being associated with additional aggressive phenotypic features including negative hormone receptor status, *HER2* amplification, and non-luminal intrinsic subtype. Our results suggest that methylation has a stronger role in the biology of certain DCIS cases than others and perhaps differences in methylation patterns could be used to classify DCIS cases in a clinically significant way. Indeed, in invasive breast cancers, a breast CpG island methylator phenotype (B-CIMP) has been described and associated with clinical outcome [40] and methylation profile has been shown to be related to intrinsic subtype [41]-[43].

Methylation of *APC*, *CDH13, RARβ* and *RASSF1A* was variably significantly associated with conventional aggressive characteristics including high nuclear grade, comedo necrosis, negative ERα status, negative PgR status, *HER2* amplification, and intrinsic subtype. The association between methylation of these genes and adverse phenotypic features in DCIS is in keeping with the role of these genes as tumour suppressor genes. APC is a component of the Wnt signaling pathway, where it forms part of a protein complex leading to the phosphorylation and degradation of β-catenin in the absence of Wnt binding [44]. CDH13 negatively controls tumour growth and invasiveness and promotes tumour neovascularization [45], while RARβ is required for the tumour suppressive effects of retinoids [46] and RASSF1A, a key player in the Hippo tumour suppressor pathway, has roles in cell cycle regulation, apoptosis and microtubule stability [47]. Indeed, in invasive breast cancer, the presence of *RARβ* methylation in both tumour and serum has been associated with poor disease-free and overall survival [48],[49], while the presence of *RASSF1A* and *APC* methylation in pre-operative serum samples predicts for poorer overall survival [50],[51], and *APC* methylation in breast cancer tissue is associated with reduced time to recurrence [17]. Similarly, a recent meta-analysis demonstrated a relationship between *RASSF1A* methylation and higher risk of relapse and poorer survival [52]. While *CDH13* methylation has not yet been directly associated with prognosis in breast cancer, *CDH13* methylation has been associated with *HER2* amplification [52] and negative PgR status [15] in invasive breast carcinoma, although the latter relationship was not confirmed in a subsequent study by the same group [53].

Unfortunately, no long-term follow-up data are available for the DCIS cases in our current study to determine whether methylation is associated with outcome in patients with DCIS. It would thus be of great interest to validate the methylation status of these genes in a large independent series with long-term follow up, annotated for known prognostic factors such as nuclear grade, margin status, and adjuvant therapy, to investigate the relationship of methylation with patient outcome.

When comparing our results with the data in the literature, we were unable to confirm the previously published associations of *RARβ*[5],[6], and *CDH1*[6] methylation with nuclear grade, higher *FOXC1*[7],[8], *GSTP1*[7],[8], and *RASSF1A*[7],[8] methylation levels with positive ERα status, higher *GSTP1* methylation levels with positive PgR status [7], and higher *RASSF1A* methylation levels with *HER2* amplification [8], although trends toward an association of *RARβ* with nuclear grade, and *RASSF1A* with positive ERα status and *HER2* amplification were also seen in our cohort (Additional file 6). These differences are likely to be due to the use of different methodologies and study populations. We have used a robust and reproducible semiquantitative method of methylation analysis, whereas the other studies have either used methylation-specific assays [5],[6] or fully quantitative methodologies, which have been performed in an exceedingly small cohorts that are likely to give rise to significant bias [7],[8]. Furthermore, our cohort consists of predominantly pure DCIS samples whereas other studies generally have examined mixed DCIS and invasive cancers [7],[8].

## Conclusions

In this study we have demonstrated a significant association between methylated genes and known prognostic features in DCIS with a candidate-gene panel approach. In particular, this is the only study focused on pure DCIS that has correlated methylation with intrinsic phenotype. We report for the first time an association of *CDH13* methylation with nuclear grade and hormone receptor status in DCIS. We have also established a new classification method based on methylation load using multiple markers. We have further shown that DNA methylation can be assessed even with small quantities of degraded FFPE DNA, enabling its use as a robust biomarker in DCIS. The next step will therefore be to investigate the role of methylation as a prognostic biomarker in a large independent cohort of pure DCIS cases with long-term follow up. It is also likely that as with invasive carcinoma some methylated genes may be of use as predictive biomarkers of hormonal therapy [54], a further avenue of investigation that warrants research effort.

## Authors’ contributions

J-MBP identified cases, prepared samples, obtained clinicopathological data, performed methylation assays, interpreted and analyzed data, and wrote the manuscript. SD and EAT interpreted data, wrote and edited the manuscript. DJB prepared sections for microdissection, constructed tissue microarrays, interpreted and analyzed data, wrote and edited the manuscript. NJ optimized and performed immunohistochemical staining of samples, wrote and edited the manuscript. AB and AH identified cases, constructed tissue microarrays, obtained clinicopathological data and constructed a database, wrote and edited the manuscript. EM, CSL, and SAO conceptualized the project, identified cases, obtained clinicopathological data and constructed a database, provided analysis, wrote and edited the manuscript. AD conceptualized the project, designed methylation assays, interpreted data, provided analysis, wrote and edited the manuscript. SBF conceptualized the project, wrote and edited the manuscript, provided project oversight and coordination, and analysis. AD and SBF share senior authorship of this manuscript. All authors read and approved the final manuscript.

## Authors’ information

Alexander Dobrovic and Stephen B Fox are joint last authors.

## Additional files

## Electronic supplementary material

Additional file 1: Patient flow in study.(XLSX 10 KB)

Additional file 2: Examples of methylation-sensitive high-resolution melting (MS-HRM patterns).(PDF 442 KB)

Additional file 3: Methylation-sensitive high-resolution melting (MS-HRM) primer sequences.(XLSX 11 KB)

Additional file 4: PCR and methylation-sensitive high-resolution melting (MS-HRM) conditions.(XLSX 12 KB)

Additional file 5: Methylation-sensitive high-resolution melting (MS-HRM) results for each sample.(XLSX 18 KB)

Additional file 6: **Methylation and ductal carcinoma** ***in situ*** **(DCIS) phenotype data.**(XLSX 23 KB)

Below are the links to the authors’ original submitted files for images.

Authors’ original file for figure 1

Authors’ original file for figure 2

## Abbreviations

AMI: average methylation index
DCIS: ductal carcinoma *in situ*
ERα: estrogen receptor alpha
FFPE: Formalin fixed, paraffin embedded
H&E: haematoxylin and eosin
HER2: human epidermal growth factor receptor-2
IHC: immunohistochemistry/immunohistochemical
MS-HRM: methylation-sensitive high-resolution melting
PgR: progesterone receptor
SISH: silver *in situ* hybridisation
TMA: tissue microarray
WGA: whole genome amplified
