# Supplementary material for: Methylation profiling of ductal carcinoma in situand its relationship to histopathological features
Source: Breast Cancer Res. 2014 Oct 21;16:423. doi: 10.1186/s13058-014-0423-9 (PMC4303108; doi:10.1186/s13058-014-0423-9)

Additional file 2: Examples of MS-HRM of A) *RASSF1A* and B-C) *TWIST1* genes in DCIS samples. A) shows sample P67 with distinct methylated and unmethylated peaks showing between 25% and 50% methylation for *RASSF1A*. B) shows sample P92 with a pattern indicative of relatively high level heterogeneous methylation for *TWIST1* and C) shows sample P69 with low level heterogeneous methylation for *TWIST1.* The heterogeneously methylated samples show a complex melting pattern with the profile extending into higher temperatures than the unmethylated control.

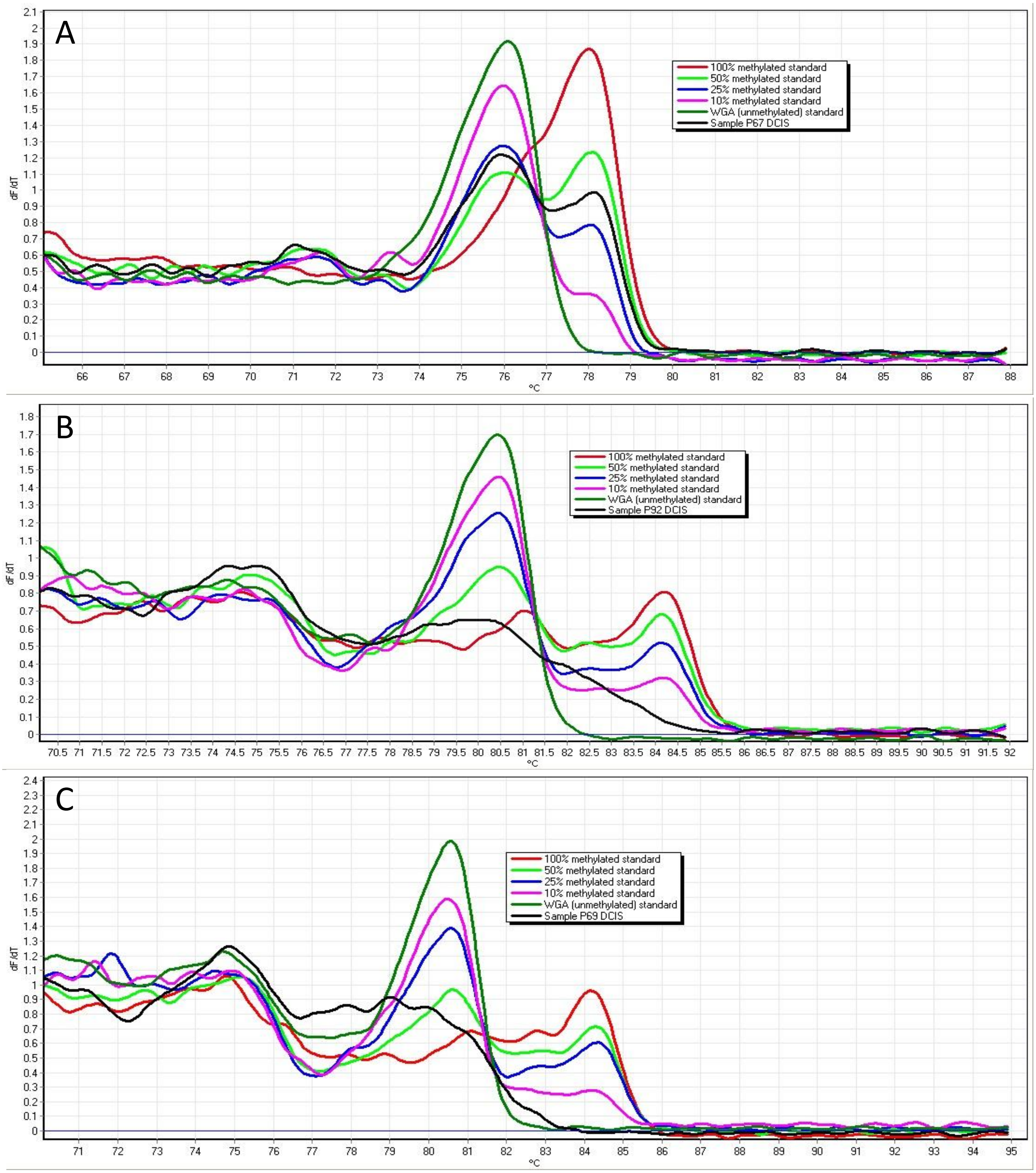

Supplement: Supplementary file 2 — Additional file 2: Examples of methylation-sensitive high-resolution melting (MS-HRM patterns).(PDF 442 KB) [file 13058_2014_423_MOESM2_ESM.pdf]
