# Supplementary figures and images for: Methylation profiling of ductal carcinoma in situand its relationship to histopathological features
Source: Breast Cancer Res. 2014 Oct 21;16:423. doi: 10.1186/s13058-014-0423-9 (PMC4303108; doi:10.1186/s13058-014-0423-9)

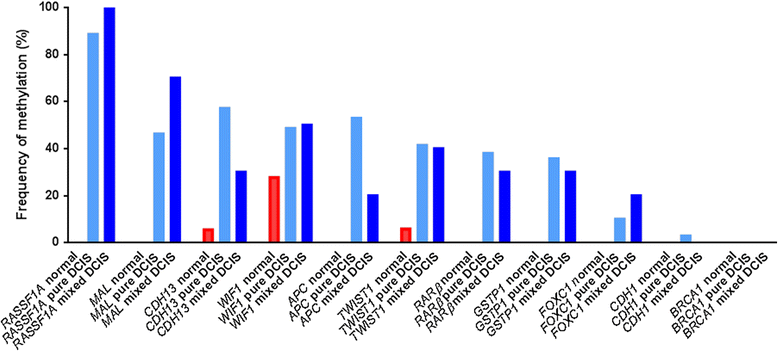

Supplement: Supplementary file 7 — Authors’ original file for figure 1 [file 13058_2014_423_MOESM7_ESM.gif]

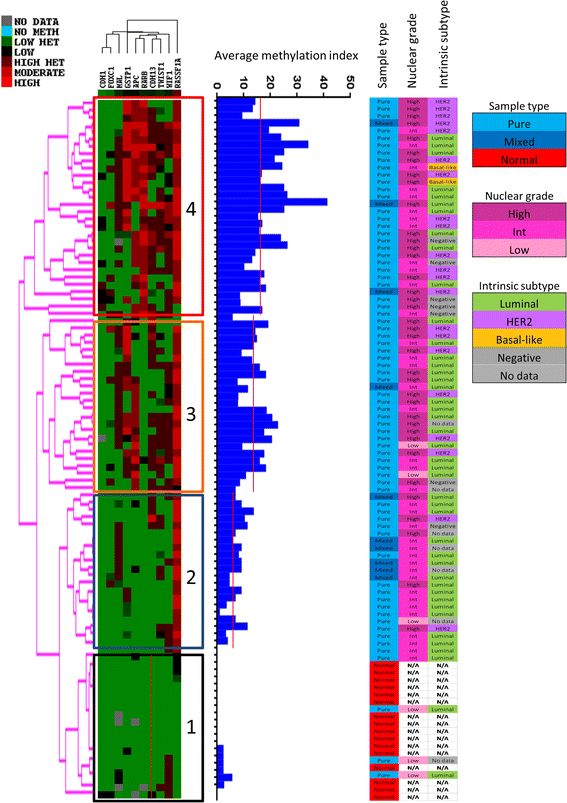

Supplement: Supplementary file 8 — Authors’ original file for figure 2 [file 13058_2014_423_MOESM8_ESM.gif]
